# Coupling phenotypic changes to extinction and survival in an endemic prey community threatened by an invasive snake

**DOI:** 10.1038/s41598-022-22583-5

**Published:** 2022-10-29

**Authors:** Julien C. Piquet, Borja Maestresalas, Marta López-Darias

**Affiliations:** Island Ecology and Evolution Research Group, Instituto de Productos Naturales y Agrobiología (IPNA-CSIC), 38206 San Cristóbal de La Laguna, Tenerife, Canary Islands Spain

**Keywords:** Herpetology, Invasive species

## Abstract

When facing novel invasive predators, native prey can either go extinct or survive through exaptation or phenotypic shifts (either plastic or adaptive). Native prey can also reflect stress-mediated responses against invasive predators, affecting their body condition. Although multiple native prey are likely to present both types of responses against a single invader, community-level studies are infrequent. The invasive California kingsnake (*Lampropeltis californiae*) a good example to explore invasive predators’ effects on morphology and body condition at a community level, as this invader is known to locally extinct the Gran Canaria giant lizard (*Gallotia stehlini*) and to notably reduce the numbers of the Gran Canaria skink (*Chalcides sexlineatus*) and the Boettger’s gecko (*Tarentola boettgeri*). By comparing a set of morphological traits and body condition (i.e. body index and ectoparasite load) between invaded and uninvaded areas for the three squamates, we found clear evidence of a link between a lack of phenotypic change and extinction, as *G. stehlini* was the single native prey that did not show morphological shifts. On the other side, surviving *C. sexlineatus* and *T. boettgeri* exhibited phenotypic differences in several morphological traits that could reflect plastic responses that contribute to their capacity to cope with the snake. Body condition responses varied among species, indicating the potential existence of simultaneous consumptive and non-consumptive effects at a community level. Our study further highlights the importance addressing the impact of invasive predators from a community perspective in order to gain a deeper understanding of their effect in native ecosystems.

## Introduction

Ecological novelty produced by invasive predators is leading to the reconfiguration of predator–prey systems worldwide, with major consequences for prey population dynamics^1,2^ and evolution^3–5^. Impacts upon native prey depend on the strength of predation pressure, as well as on prey capacity to successfully coexist with novel predators^6,7^. Theory predicts that when prey lack the ability to evolve, they are led to extinction^6–8^, a fact often linked to the absence of a predator–prey common evolutionary history^8,9^. However, empirical studies corroborating this idea are lacking as species driven to extinction are usually absent or will soon be. Alternatively, prey can survive by means of exaptation—i.e., characters that evolved for other usages (or neutral characters) and later coopted for their current role^10^—or by phenotypic changes allowing them to avoid predation^6–9^. These changes generally affect four main aspects of native species biology^6^ including behavior^11,12^, physiology^13,14^, life history traits^5,15^ or morphology^3–5,16,17^. Shifts on morphological traits in particular have been the focus of extensive research that generally consists in single-prey studies^3–5,16,17^, although invasive predators are generalists that affect multiple native species^18–20^. In this context, first stages of predator invasions are an ideal scenario to study the phenotypic responses (or their lack) of prey communities and link them to prey extinction or survival.

Predator–prey interactions happen through a series of sequential steps that include predator–prey activity overlapping in space and time and prey failing to avoid and escape predators^21^. Both prey exaptation and phenotypic changes can contribute to end this predation sequence at any step^6,7^. For instance, pre-existing patterns of diel activity or habitat use in prey can minimize the overlap with invasive predators^22^. Changes in morphology, particularly in those traits associated with prey climbing and burrowing capacities or sprint speed, performance, or endurance^23–28^ can prevent the predation sequence to progress through the final steps of predator avoidance and escape^3,4,16,29^. Nevertheless, predators can also induce heavy stress responses on prey^29^ that non-consumptively affect body condition^30^ and potentially impact long-term prey survival^31^. Although these changes are key to understand prey survival under the novelty caused by invasive predators, evidence on how they interact is mostly absent.

The recent invasion of the California kingsnake (detected in Gran Canaria *c.* 25 years ago^32^) provides an ideal example to evaluate real-time morphological and body condition responses to invasive predators at a community level. This invasive predator is a medium-sized colubrid snake—adult snout-to-vent length (SVL) range: 61–130 cm^33^—native to western USA and northwest Mexico^34^, whose diet in the island mainly relies on the unique three endemic reptile species—the Gran Canaria giant lizard (*Gallotia stehlini*), the Gran Canaria skink (*Chalcides sexlineatus*), and Boettger’s wall gecko (*Tarentola boettgeri*)^35^. Predation pressure is high for the entire reptile community, leading to disparate responses in the three species that are particularly noticeable in areas that have been invaded earlier^36^. While *G. stehlini* ends up locally extinct (over 95% reduction in numbers), *L. californiae* reduces *C. sexlineatus* and *T. boettgeri* numbers to 82.8% and 52.1%, respectively^36^. The severity of this impact is probably linked to the lack of a co-evolutionary history between these reptiles and predatory snakes^36^, as these predators have never been present in the island^37,38^—before *L. californiae* arrived, reptile main predators were native birds of prey^39^ and invasive feral cats^40^. However, differences in population responses to *L. californiae* might relate to differing morphology and ecological habits of *G. stehlini*, *C. sexlineatus,* and *T. boettgeri*, which overlap to a different extent with that of the invasive predator. *Gallotia stehlini* is a large-sized (≤ 28 cm SVL), big-headed lacertid lizard (max: 4.85 × 4.61 cm, W × H; authors’ own data), with diurnal and surface-dwelling habits^41^, showing main activity on warmer times of the day and year^36,42^. *Chalcides sexlineatus* is a medium-sized (≤ 9.3 cm SVL), diurnal, and epigeal or semi-fossorial skink^43^, and *T. boettgeri* is a small-sized (≤ 7.5 cm SVL), nocturnal, and scansorial gecko that perches upside-down under rocks during daytime^44,45^. Conversely, *L. californiae* is a diurnal-crepuscular, fossorial^33^, wide-searching predator that consumes prey with similar diel habits to its own^46^, swallowing them whole, so prey consumption is constrained by snake gape size^47^. In this context, we performed a field study to explore morphology and body condition shifts in the whole reptile community with the aim to delve into the consequences of the different impacts recorded for the three species. We hypothesized that *G. stehlini* constitutes a real-time example of species extinction that is coupled with a lack of phenotypic response. On the other hand, we expect that the survival of *C. sexlineatus* and *T. boettgeri* to the novel predator is linked to phenotypic changes, particularly with traits that favor predator avoidance (i.e., burrowing for *C. sexlineatus* or clinging for *T. boettgeri*) or escape behavior (i.e., running for both species). Finally, we expect that *L. californiae* will worsen body condition on all the three endemic reptiles, potentially through fear-mediated effects. In a wider perspective, this study provides additional evidence on a sort of invasive predator impacts that is less extensively described^48^.

## Methods

### Sampling sites, trapping method and sampling numbers

**Figure 1:**
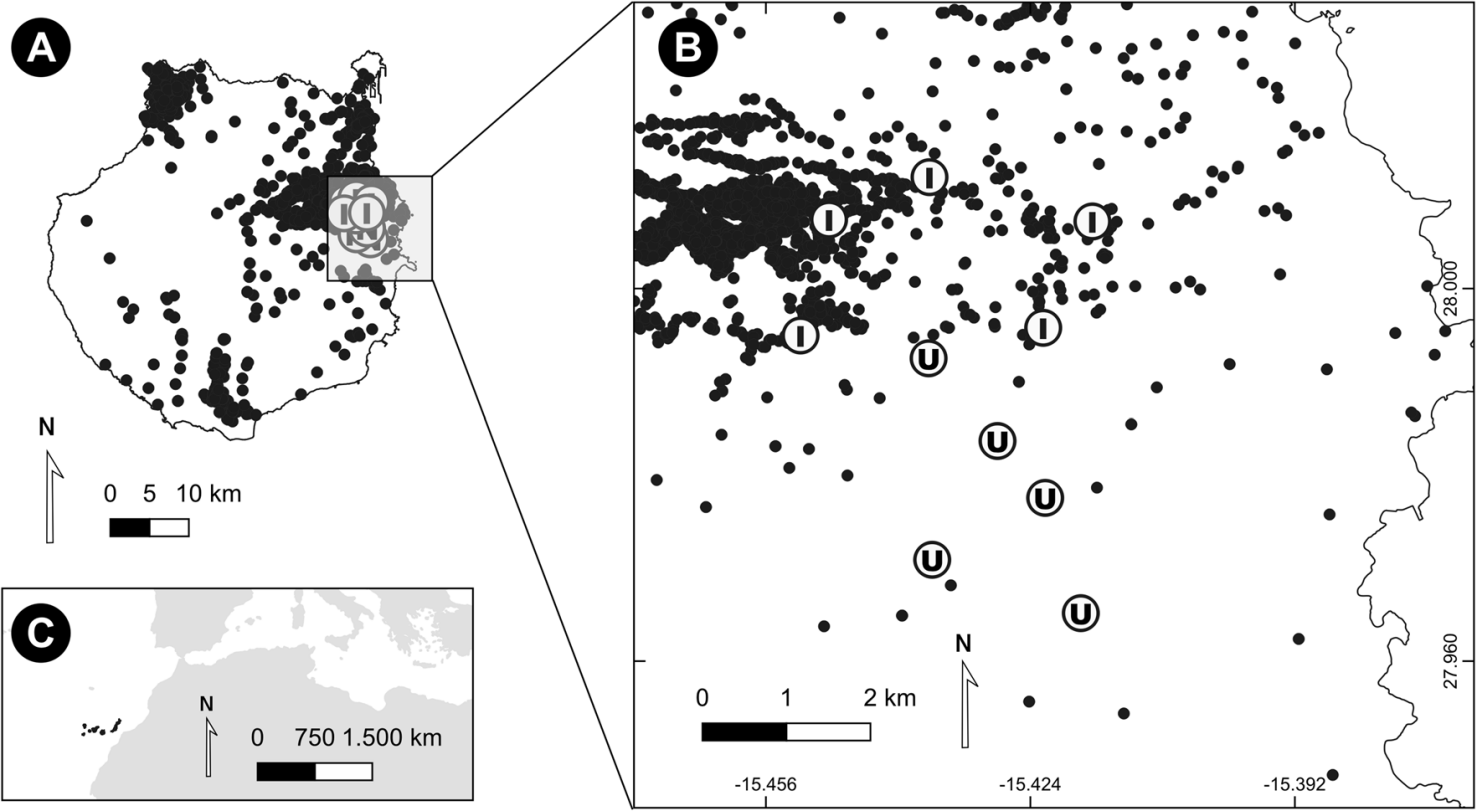
Location of the study area (**A**), sampling sites in Gran Canaria (**B**), and the Canary Islands location (**C**). Sampling sites within the area invaded by *Lampropeltis californiae* are identified with an I (invaded), whereas those away from snake records are marked with an U (uninvaded). Black dots represent snake records from 2009 to 2020 (provided by GESPLAN S.A.). Map created using QGIS 3.20 (http://www.qgis.org).

To measure differences in morphology and body condition on the three endemic reptiles as a response to *L. californiae*, we designated a total of 10 sampling sites, five in invaded and five in uninvaded areas (Fig. 1). We selected sites to include recently invaded areas, were all prey were still present. To minimize the potential effect of biotic and abiotic variables on phenotype, we placed all sites in a small area (< 19 km^2^) and within a narrow altitudinal range (from 178 to 283 m a.s.l.), all of them vegetated with native coastal shrubland^49^ interspaced with formerly cultivated areas. To account for the snake home range (Maestresalas et al. under review)﻿, we located uninvaded areas at least 200 m away from the closest record of *L. californiae* according to http://www.lifeampropeltis.com (Fig. 1).

To increase capture efficiency of the endemic herpetofauna, we performed fieldwork during the warmest months of the year (March–November)^50^, which matches the period when all study species are more frequently active. In the particular case of *G. stehlini*, we carried out captures only on sunny and warm days, as temperature influences its activity^36^. To avoid recaptures, we performed a single capture session per site and species, retained captured reptiles in individual cloth bags, and measured all of them at the end of each session. We noted down the capture location of all individuals with Google©MyMaps and released them exactly at the same place within the next day after their capture. We sampled 143 adult *G. stehlini* (October–November 2019; see Table S1.1 for more details) by noosing or trapping with pitfall (30 × 40 × 50 cm, L × W × D), PVC funnel (11 × 110 cm, H × L, with an inner entrance of 3 × 3 cm), and box traps (22.5 × 48 × 48, H × L × W^51^), baited with tomatoes and sardines, and located them as to minimize sun exposure. We collected 102 adult *C. sexlineatus* (March–April 2021) and 247 adult *T. boettgeri* (May 2019) (Table S1.1) through active searches beneath rocks that act as refuges during daylight^41,43,44^.

### Morphology and body condition

**Figure 2:**
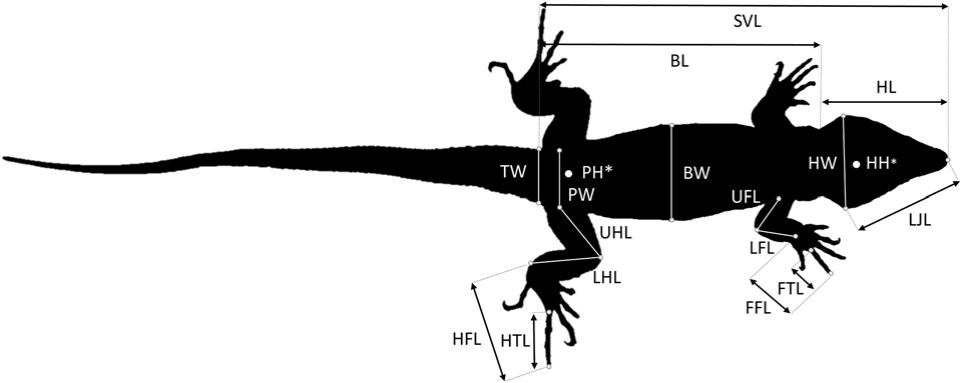
Set of morphological measurements used for the Gran Canaria giant lizard (*Gallotia stehlini*), the Gran Canaria skink (*Chalcides sexlineatus*) and Boettger’s wall gecko (*Tarentola* *boettgeri*). Snout-vent length (SVL), from the tip of the snout to the posterior tip of the anal scale; head length (HL), from the tip of the snout to the posterior part of the parietal scales; head width (HW), widest point of the head at the level of the jugal bones; head height (HH), highest part of the head, posterior to the orbits; lower jaw length (LJL), from the tip of the lower jaw to the back of the retroarticular process; longest fore toe length (FTL), from the base of the longest toe to the base of the claw; forefoot length (FFL), from the proximal end of the metacarpus to the distal end of the longest toe; lower forelimb length (LFL), from the elbow to the proximal end of the metacarpus; upper forelimb length (UFL), from the insertion of foreleg into body to the elbow; longest hind toe length (HTL), from the base of the longest toe to the base of the claw; hind foot length (HFL), from the proximal end of the metacarpus to the distal end of the longest toe; lower hind limb length (LHL), from the knee to the proximal end of the foot; upper hind limb length (UHL), from the insertion to the body to the knee; pelvis height (PH), height of the body immediately anterior to the hind legs; pelvis width (PW), width of the body immediately anterior to hind legs; tail width (TW), widest portion of the tail; body width (BW), widest portion of mid-body; body length (BL), from the shoulders to the pelvis; LJL was taken only for *G. stehlini—*due to the difficulty in obtaining precise measurements for this trait in *C. sexlineatus* and *T. boettgeri*—whereas UFL, FFL, UHL, HFL, PH, PW, TW, BW and BL were only noted for *C. sexlineatus*, as these traits are related to burrowing capacity in skinks^23^.

We used a plastic ruler (± 0.1 cm) to measure SVL in each individual, and a digital caliper (± 0.01 mm, OWIM GmBH & Co. KG, Neckarsulm, Germany) to characterize head, limbs, and body traits related with behavioral attributes (sprint, burrowing and clinging ability)^23–28^ that contribute to predator avoidance and escape (Fig. 2). To avoid observer bias, a single observer measured each species (JCP *G. stehlini* and *C. sexlineatus*, and MLD, *T. boettgeri*). We determined sex of each individual by everting hemipenises for *G. stehlini* and *C. sexlineatus* and by shining a light dorsally to the base of the tail to visualize them in *T. boettgeri*^52^. Additionally, we quantified the number of lamellae of *T. boettgeri*—the single pad-bearing squamate on Gran Canaria—by photographing the longest fore toe, counting their lamellae using a common photo software, and averaging them for later analyses^53^. Lastly, we evaluated body condition in each species with the scaled mass index following the formula described by Peig and Green^54^, and by quantifying individual ectoparasite loads. This index is appropriate as it accounts for morphological differences in sexually dimorphic species^55^, such as the study ones^43,56–58^. To calculate the scaled mass index, we measured body mass with a digital weight scale (± 0.1 g, Ohaus, Nänikon, Switzerland). We also calculated mite prevalence (number of individuals infected in each species sample) and abundance (number of mites per individual)^59^ for the three species, after counting mites with a magnifying glass (30X); ectoparasites were absent in *C. sexlineatus* (see Table S1.1). Mites were *Ophionyssus setosus* for *G. stehlini* and *Geckobia* spp. for *T. boettgeri*^60,61^. To avoid bias due to different observers, JCP performed mite counts.

### Data analysis

To detect potential outliers, we first performed Rosner tests^62^ on log_10_-SVL, all log_10_-transformed traits regressed against log_10_-SVL, scaled mass index and mite abundance. We later removed outliers potentially attributed to observer errors^63^.

We used GLMMs (except for lamellae counts) performed with *glmmTMB* package^64^ to explore differences in morphological and body condition traits for each species, adding *log*_*10*_*-SVL* as a covariate in morphological models. We included *snake presence* (invaded *vs*. uninvaded sites), *sex* and *snake presence* × *sex* as fixed factors, with *snake presence* and its interaction with *sex* being the main effects of interest in our study. We entered *site* as a random factor. We assigned Gaussian distribution to all response variables, except for mite prevalence and abundance, which had a binomial and negative binomial distribution with a quadratic parametrization term (after checking the lower fit of Poisson distribution), respectively. To account for the heterogeneity of the variance, we also included *site* as a dispersion factor for the SVL model (along with *sex* in the case of *G. stehlini* and *C. sexlineatus*), all remaining morphological models of *G. stehlini* and *T. boettgeri*, and all models for body condition variables (along with *sex* for the scaled mass index). We visually inspected model residuals in *DHARMa* package^65^, retrieved model main effects using type-II Wald Chi-square tests^66^ via ‘Anova’ function, and obtained the significance of the estimates using *emmeans* package^67^—we adjusted *P* values for multiple comparisons using false discovery rates^68^. Finally, due to discrete and leptokurtic distribution of *T. boettgeri* lamellae counts and to avoid pseudoreplication, we compared mean values of this trait per site between invaded and uninvaded sites using Kruskal–Wallis tests for sexes separately (after verifying no association existed between log_10_-SVL and lamellae count; data not shown).

We ran all analyses in R 4.1.2^69^. All results are presented as mean ± SD, except stated otherwise.

### Ethical statement

We had all necessary authorizations to perform our research (Cabildo de Gran Canaria No. 003/18 and No. 397/19, and Gobierno de Canarias No. 2018/728 and No. 2020/12858). We were provided with ethical clearance by the Canarian Government No. 159/2021. We carried out all experiments in compliance with the ARRIVE guidelines (https://arriveguidelines.org), performing all methods in accordance with relevant guidelines and regulations.

## Results

### Snake influence on endemic reptile morphology

**Figure 3:**
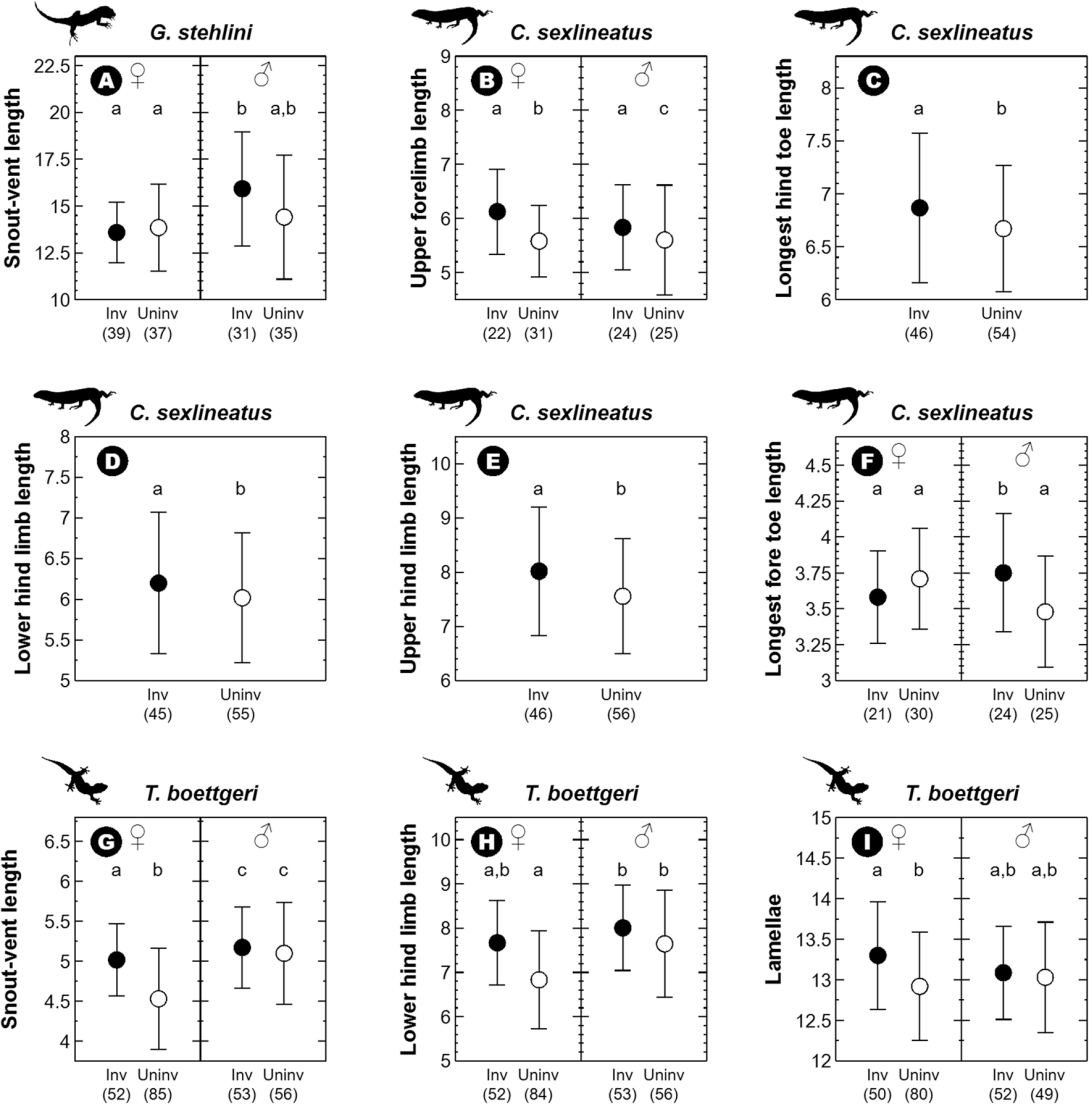
Mean and SD of the morphological traits of adult females (♀) and males (♂) of Gran Canaria giant lizards (*Gallotia stehlini*) (**A**), Gran Canaria skinks (*Chalcides sexlineatus*) (**B**–**F**) and Boettger’s wall geckos (*Tarentola boettgeri*) (**G**–**I**), that presented significant differences between sites invaded (inv; black) and uninvaded (uninv; white) by the California kingsnake (*Lampropeltis californiae*). Sample size for each trait—i.e., number of individuals captured for which each trait was measured, excluding values attributed to observer error—is indicated between parentheses below each figure. Significant differences are signaled with different letters. All morphological measurements are in mm, except the SVL (cm) and the number of toepad lamellae (no. lamellae). All silhouettes come from phylopic.org or the free repository of the Government of the Canary Islands.

SVL was the only trait in *G*. *stehlini* that was influenced by *L. californiae*, females being smaller than males only in invaded sites (*snake presence* × *sex*: ${\upchi }_{{1}}^{{2}}$ = 10.25, *P* = 0.001; *t*_128_ = − 4.45, *P* < 0.001; Fig. 3A; see Table S1.1 for mean values and Table S2.1 for complete model results).

The effect of the snake upon *C. sexlineatus* appeared only for limb traits (see Table S2.2). *Snake presence* explained that skinks showed longer upper forelimb, longest hind toe, lower hind limb, and upper hind limb in invaded sites (*P* < 0.01 in all cases; Fig. 3B–E; Tables S1.1 and S2.2). An effect of *snake presence* × *sex* appeared for the longest fore toe, which was longer in invaded sites only for males (*snake presence* × *sex*: ${\upchi }_{{1}}^{{2}}$ = 7.03, *P* = 0.008; *t*_93_ = − 3.16, *P* = 0.002), and the upper forelimb, for which both sexes differed only in uninvaded sites (*snake presence* × *sex*: ${\upchi }_{{1}}^{{2}}$ = 6.66, *P* = 0.010; *t*_95_ = − 2.58, *P* = 0.012; Fig. 3B,F; Tables S1.1 and S2.2).

*Tarentola boettgeri* individuals were larger in invaded than in uninvaded sites (*snake presence*: ${\upchi }_{{1}}^{{2}}$ = 9.57, *P* = 0.002), with females showing higher values of SVL in invaded areas (*snake presence* × *sex*: ${\upchi }_{{1}}^{{2}}$ = 6.49, *P* = 0.011; *t*_231_ = − 3.97*, P* < 0.001; Fig. 3G; Tables S1.1 and S2.3). Moreover, females had shorter lower hind limb length than males only in uninvaded sites (*snake presence* × *sex*: ${\upchi }_{{1}}^{{2}}$ = 5.96, *P* = 0.015; invaded: *t*_229_ = − 1.24*, P* = 0.215; uninvaded: *t*_229_ = 2.20*, P* = 0.029; Fig. 3H; Table S1.1 and S2.3). Finally, females showed significantly higher number of lamellae in invaded than in uninvaded areas (Kruskal–Wallis: ${\upchi }_{{1}}^{{2}}$ = 3.94, *P* = 0.047; Fig. 3I; Table S.1.1 and S.2.3).

### Snake influence on body condition

**Figure 4:**
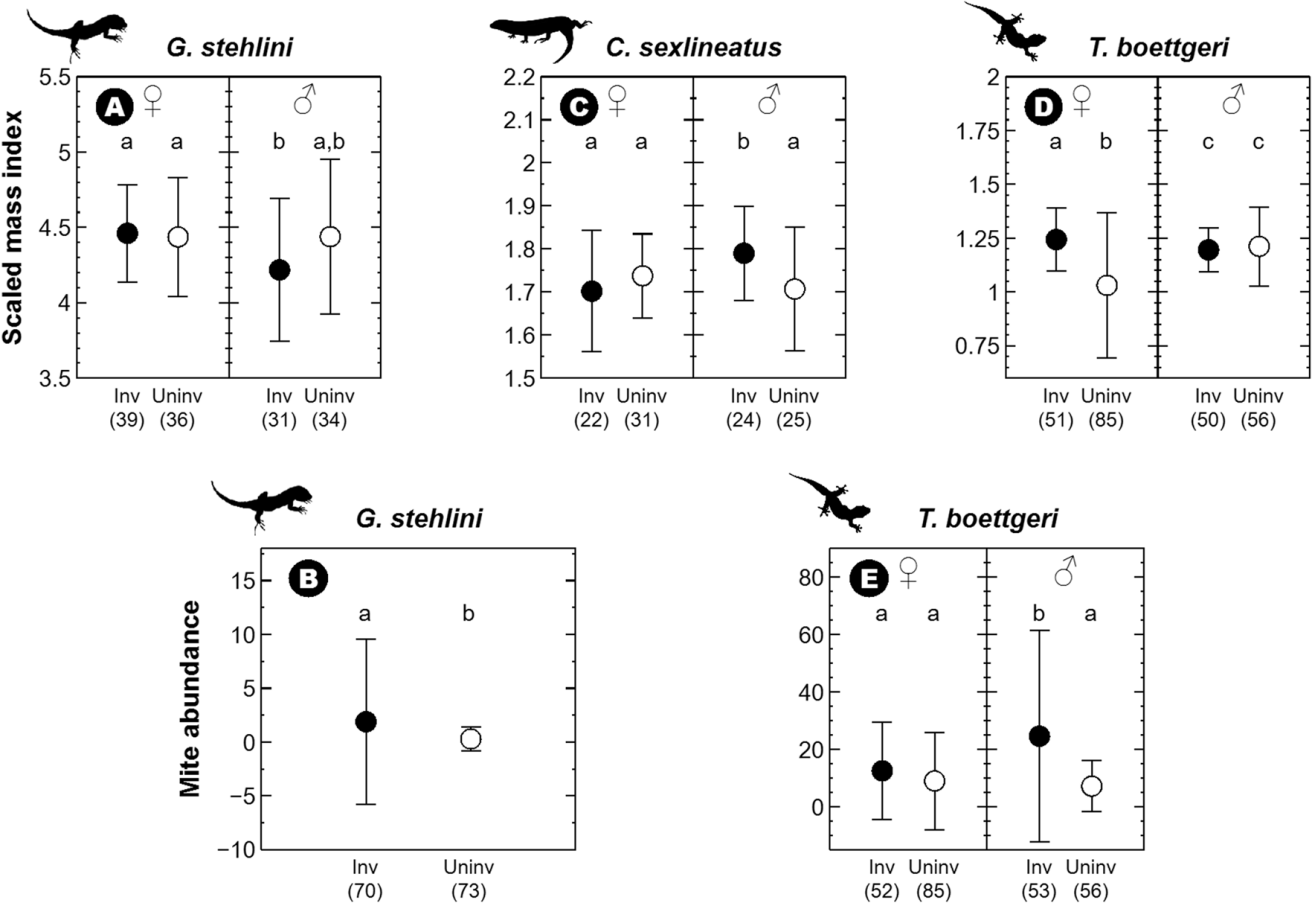
Mean and SD (error bars) of the scaled mass index^53^ and mite abundance in adult females (♀) and males (♂) of Gran Canaria giant lizards (*Gallotia stehlini*) (**A**,**B**), Gran Canaria skinks (*Chalcides sexlineatus*) (**C**) and Boettger’s wall geckos (*Tarentola boettgeri*) (**D**,**E**) in sites invaded (inv; black) and uninvaded (uninv; white) by the California kingsnake (*Lampropeltis californiae*). Sample size for each trait—i.e., number of individuals captured for which each trait was measured, excluding values attributed to observer error—is indicated between parentheses below each figure. Parasite loads are not shown for *C. sexlineatus* due to the absence of parasites in this species. Significant differences are signaled with different letters. All silhouettes come from phylopic.org or the free repository of the Government of the Canary Islands.

Male *G. stehlini* were thinner than females with respect to their body size in invaded (*t*_126_ = 3.21, *P* = 0.002) but not in uninvaded sites (*t*_126_ = 0.30, *P* = 0.764) (*snake presence* × *sex*: ${\upchi }_{{1}}^{{2}}$ = 4.19, *P* = 0.041; Fig. 4A; Tables S1.1 and S2.1). Mite abundance was significantly higher in invaded sites regardless of sex (*snake presence*: ${\upchi }_{{1}}^{{2}} { }$ = 6.09, *P* = 0.014; Fig. 4B; Tables S1.1 and S2.1).

*Chalcides sexlineatus* scaled mass index was affected by the presence of *L. californiae*, males being thicker for their size in invaded sites (*snake presence* × *sex*: ${\upchi }_{{1}}^{{2}}$ = 5.16, *P* = 0.023; *t*_86_ = − 2.26, *P* = 0.027; Fig. 4C; Tables S1.1 and S2.2).

Female *T. boettgeri* were thicker for their size in invaded sites (*snake presence* × *sex*: ${\upchi }_{{1}}^{{2}}$ = 23.85, *P* < 0.001; *t*_226_ = − 3.84, *P* < 0.001; Fig. 4D; Tables S1.1 and S2.3). Males had significantly more mites in invaded sites (*snake presence* × *sex*: ${\upchi }_{{1}}^{{2}}$ = 7.25, *P* = 0.008; *t*_231_ = − 2.57, *P* = 0.011; Fig. 4E; Tables S1.1 and S2.3).

## Discussion

This study helps enlighten one of the most accepted but not so proven ecological and evolutionary theories: the lack of adaptation to changes is linked to the extinction of species and vice versa. By analyzing the first stages of the snake invasion, we found clear evidence of a lack of morphological shifts only in the native reptile that is facing extinction, *G. stehlini*^37^. Conversely, *C. sexlineatus* and *T. boettgeri*, which are both surviving to a different extent to the invasive predator^37^, presented relevant shifts in morphological traits related with behavioral attributes^24–29^ that can contribute to predator avoidance and escape. In addition, the presence of invasive predators was associated with phenotypic shifts differing across prey traits (both morphology and body condition) within a single community, which underscores how important is to study entire communities to fully understand the effect of invasive predators.

*Gallotia stehlini* is the species facing the highest impact from *L. californiae* predation pressure^37^, yet phenotypic differences in this prey were only patent in body length, a trait intimately related to age in reptiles^70^. Older *G. stehlini* attain considerable body sizes and develop particularly big heads^71^, while exhibiting aggressive antipredator responses^72^. Since snakes consume ectothermic prey alive^73^, smaller individuals with smaller heads are thus more easily subdued and consumed. For instance, following King gape index^47^, less than one third of adult *G. stehlini* from this study had a cross-sectional area at head level that would impede their consumption by *L. californiae* (authors’ data). In this sense, although males and females in *G. stehlini* can attain similar body lengths^74^, males show relatively wider heads for their size^71^. *Lampropeltis californiae* gape size could thus allow the consumption of large females but not that of males of the same size, leading to the observed differences in SVL between sexes only in invaded sites. Complementary, this could also be the consequence of the higher proportion of males at the upper end of body size and thus head width range in *G. stehlini* populations—e.g., from all individuals in our study presenting head dimensions over *L. californiae* gape size, almost 60% were males. All of this suggests that *L. californiae* impact probably begins with the removal of smaller individuals with narrower heads—i.e., younger lizards, smaller males and most females. Considering *G. stehlini* is a long-lived lizard that lives up to 10–11 years in the wild^73^, which exceeds the time since the snake arrival in some of our study sites, persisting *G. stehlini* populations in the invaded areas are likely constituted by a disproportionate number of few large and old individuals, most of which are probably males—supported by our own observations in the field. Therefore, unless populations in invaded areas show strong immigration rates, which is apparently not the case, they will likely become extinct. This is confirmed by the absence of *G. stehlini* in areas invaded for a longer period and some areas recently invaded by *L. californiae*^36^, which indicates that snakes cause swift population abatement. In this context, adaptive responses against *L. californiae* are improbable in *G. stehlini*, as these often require native prey being able to cope with the invasive predator to a certain extent^6,7,75^. The lack of differences between invaded and uninvaded sites in the remaining morphological traits support this interpretation and suggest that *G. stehlini* is failing to adapt to the invasive snake. Overall, our findings regarding *G. stehlini* coincide with the notion that when exaptation and adaptive responses against invasive predators fail, native prey are doomed with extinction^6,7,75^.

Unlike *G. stehlini*, *C. sexlineatus* and *T. boettgeri* have undergone notable phenotypic changes. To avoid predation, prey need to break the predation sequence by limiting spatial and temporal overlap with the predator, avoid it or successfully escape when attacked^21^, which can be attained through the expression of plastic or rapid adaptive changes in phenotype^3,4,6,7^. We found that *C. sexlineatus* exhibited longer limbs and toes in the presence of the snake, whereas female *T. boettgeri* had lower hind limb lengths of comparable size to that of males—notice that lower hind limbs of females were shorter than those of males in uninvaded sites—and higher number of lamellae in the invaded areas. Limb morphology is tightly related to locomotor performance, sprint speed, and endurance^27–29,75^, thus these changes might be favoring escape capacity of both species. Additionally, lamellae counts are closely related to habitat use and perching behavior in other pad-bearing lizards^26,76,77^, as well as sprint speed^26^, so that differences observed in *T. boettgeri* might be contributing to both predator avoidance and escape. Interestingly, *T. boettgeri* also exhibited an upward shift in female body size, which is unlikely caused by size-based selection since *T. boettgeri* is a small gecko^41^ that lies in the range of species consumed by *L. californiae*^35,46^, and whose cross-sectional area is much smaller than that of other *L. californiae* prey. Therefore, this shift could reflect a higher vulnerability to predation of younger, less experienced and subordinate adults^12,44,78,79^. The fact that these changes were only observed for females can also be related to fieldwork being performed during the reproductive season—which lasts from March to August^80^—when females are more vulnerable^81,82^.

All observed morphological changes in *C. sexlineatus* and *T. boettgeri* could result from the removal of individuals with certain morphological characteristics from current populations, leading to short-term differences in trait distribution. However, considering that snakes arrived to some of our study sites decades ago, our results are more likely to reflect medium-to-long term processes. Among these, plastic changes are potentially more plausible than genetic responses, as phenotypic plasticity is more likely when prey lack of co-evolutionary history with novel predators, their populations are reduced and genetically depauperate^6,7^, and less than 25 generations have occurred since the beginning of the invasion^83^, all of which applies to both species. Considering that these two species are being drastically reduced in invaded areas^36^, two potential future scenarios could be expected^6^. First, the ancestral phenotype might be moving to a new phenotypic optimum that will finally allow the species to recover their densities and genetically assimilate the observed changes. Second, they might be trapped into displaying maladaptive phenotypes that could either lead to a more adaptive stage or to extinction. However, this provides correlative evidence alone, thus further research should experimentally confirm that phenotypic shifts actually represent adaptive responses with fitness consequences, and determine which are the potential long-term consequences for *C. sexlineatus* and *T. boettgeri* populations. On the other hand, phenotypic changes in *C. sexlineatus* and *T. boettgeri*, might have been facilitated by the existence of exaptation traits. The existence of appropriate antipredator defenses—e.g., small body size and fossoriality in *C. sexlineatus* and clinging capacity in *T. boettgeri*—may be responsible for the lower population reduction in these species compared to *G. stehlini*, allowing them to express phenotypic shifts. Although the present study approaches solely prey morphology, phenotypic shifts in *C. sexlineatus* and *T. boettgeri* may have also affected their behavior and the expression of appropriate antipredator responses, as prey that are able to avoid extinction should not remain naïve^74^. In any case, any potential expectation regarding the expression of phenotypic shifts and their long-term consequences could be altered by other predator–prey interactions involving other species (i.e., invasive rodents, feral cats, birds of prey), even under the simplified context of islands^84^.

Invasive predators are known to cause stress-mediated responses^85^, ultimately leading to reduced body condition in native prey^30^. However, predators can also selectively prey on and remove individuals with poorer body condition^86–89^, expectably causing the opposite effect in the population. Our study provides correlative evidence for both processes. First, male *G. stehlini* decreased their scaled mass index with respect to the population average in invaded sites, leading to intersexual differences that were absent in uninvaded sites. This result is probably a consequence of the higher proportion of males averting snake predation due to their bigger head size, as outlined previously. Females could face similar stress responses, but given the severity of snake predation for *G. stehlini*, non-consumptive effects are likely outweighed. *Gallotia stehlini* and male *T. boettgeri* also showed increased parasite loads, which is usually related to increased stress^90,91^. These results coincide with predictions regarding non-consumptive effects, and matches results from previous research^92^, suggesting that invasive predators can induce stress-mediated responses even in prey that lack of co-evolutionary history with them. However, male *C. sexlineatus* and female *T. boettgeri* had greater scaled mass index values in invaded sites, suggesting also that individuals with lower body condition—showing potentially less effective antipredator behavior^93,94^—were being selectively preyed on by *L. californiae.* Both body condition and parasite load are extremely variable traits and the impact of predation pressure on them is highly context- and species-dependent^95^, often resulting in unpredictable patterns and outcomes^30,96,97^. Thus, interpreting this type of results is always a complex task, particularly from a community perspective, where species can exhibit different and contrasting responses against the same predator. In spite of that, the detection of differences in both parameters between invaded and uninvaded sites is already indicative of an influence of *L. californiae*, although further studies would be needed to uncover the exact mechanisms involved in each of the three species.

Species phenotype is faceted by multiple processes, including predation^3–5^, competition^53^, human-induced disturbance^26^, natural dynamics^98,99^, and habitat features^26,76,100^, making the interpretation of phenotypic shift a complex endeavor, especially from a community perspective. However, the present study reports correlative evidence of different phenotypic shifts co-occurring in a single prey community in an extremely small geographical area and a uniform ecological and evolutionary context, highlighting the potential of understanding these complex processes when analyzing a community as a whole. In addition, our study illustrates how extinction, previously described^36^, is coupled with the lack of phenotypic change in one species of the community, whereas those two able to cope with novel predators show morphological shifts. Body condition responses against a same invasive predator can vary or even show opposite trends among different prey within the same community, highlighting the usefulness of evaluating these responses also from a community perspective. Finally, we offer evidence for a link between the presence of invasive snakes and phenotypic changes in a whole community of endemic prey, which has the potential to cascade into population, community or ecosystem-wide impacts^101^.

## Supplementary Information

Supplementary Information.

## Data Availability

All data and R code for the analyses are available in Figshare under 10.6084/m9.figshare.17167313.v3.
