# Supplementary material for: Coupling phenotypic changes to extinction and survival in an endemic prey community threatened by an invasive snake
Source: Sci Rep. 2022 Oct 29;12:18249. doi: 10.1038/s41598-022-22583-5 (PMC9617863; doi:10.1038/s41598-022-22583-5)
Supplement: Supplementary file 1 — Supplementary Information. [file 41598_2022_22583_MOESM1_ESM.docx]

COUPLING PHENOTYPIC CHANGES TO EXTINCTION AND SURVIVAL IN AN ENDEMIC PREY COMMUNITY THREATENED BY AN INVASIVE SNAKE

Julien C. Piquet^1^, Borja Maestresalas^1^, Marta López-Darias^1*^

^1^ Island Ecology and Evolution Research Group, Instituto de Productos Naturales y Agrobiología (IPNA-CSIC), 38206 La Laguna, Tenerife, Canary Islands, Spain.

^*^ Correspondence author: mdarias@ipna.csic.es; Tel.: +34 922474328

# Supplementary Information S1

**Table S1.1.** Morphological and body condition variables (Mean ± SD) of adult females and males of the Gran Canaria giant lizard (*Gallotia stehlini*), the Gran Canaria skink (*Chalcides sexlineatus*) and Boettger’s wall gecko (*Tarentola boettgeri boettgeri*) in sites invaded and uninvaded by the California kingsnake (*Lampropeltis californiae*). The sample size used in the analysis of each character is shown in each case. Morphological measurements are (see Figure 2 in the main text) snout-vent length (SVL), from the tip of the snout to the posterior tip of the anal scale; head length (HL), from the tip of the snout to the posterior part of the parietal scales; head width (HW), widest point of the head at the level of the jugal bones; head height (HH), highest part of the head, posterior to the orbits; lower jaw length (LJL), from the tip of the lower jaw to the back of the retroarticular process; longest fore toe length (FTL), from the base of the longest toe to the base of the claw; forefoot length (FFL), from the proximal end of the metacarpus to the distal end of the longest toe; lower forelimb length (LFL), from the elbow to the proximal end of the metacarpus; upper forelimb length (UFL), from the insertion of foreleg into body to the elbow; longest hind toe length (HTL), from the base of the longest toe to the base of the claw; hind foot length (HFL), from the proximal end of the metacarpus to the distal end of the longest toe; lower hind limb length (LHL), from the knee to the proximal end of the foot; upper hind limb length (UHL), from the insertion to the body to the knee; pelvis height (PH), height of the body immediately anterior to the hind legs; pelvis width (PW), width of the body immediately anterior to hind legs; tail width (TW), widest portion of the tail; body width (BW), widest portion of mid-body; body length (BL), from the shoulders to the pelvis; scaled mass index, following the formula by Peig & Green^1^; mite prevalence and mite abundance–*i.e.*, *Ophionyssus setosus* on *G. stehlini* and *Geckobia* spp. on *T. boettgeri*. All measurements are presented in mm, except for SVL (cm), lamellae (number), scaled mass index, mite prevalence (%), and mite abundance (number).

| **Traits** | ***Gallotia stehlini*** | | | | | | ***Chalcides sexlineatus*** | | | | | | ***Tarentola boettgeri*** | | | | | |
| --- | --- | --- | --- | --- | --- | --- | --- | --- | --- | --- | --- | --- | --- | --- | --- | --- | --- | --- |
|  | **Female** | | | **Male** | | | **Female** | | | **Male** | | | **Female** | | | **Male** | | |
|  | Invaded | Uninvaded | *N* | Invaded | Uninvaded | *N* | Invaded | Uninvaded | *N* | Invaded | Uninvaded | *N* | Invaded | Uninvaded | *N* | Invaded | Uninvaded | *N* |
| SVL | 13.58 ± 1.61 | 13.85 ± 2.32 | 39/37 | 15.92 ± 3.04 | 14.41 ± 3.31 | 31/35 | 7.42 ± 0.93 | 7.63 ± 0.73 | 22/31 | 7.24 ± 0.85 | 7.23 ± 0.97 | 24/25 | 5.02 ± 0.45 | 4.53 ± 0.63 | 52/85 | 5.17 ± 0.51 | 5.10 ± 0.64 | 53/56 |
| HL | 32.15 ± 4.27 | 32.69 ± 6.12 | 39/36 | 40.46 ± 8.92 | 35.50 ± 8.98 | 29/34 | 10.89 ± 1.01 | 11.01 ± 0.98 | 22/31 | 11.07 ± 1.27 | 10.96 ± 1.42 | 24/25 | 14.51 ± 1.16 | 13.44 ± 1.51 | 51/82 | 15.24 ± 1.34 | 15.05 ± 1.76 | 53/55 |
| HW | 14.97 ± 1.86 | 15.17 ± 2.69 | 39/36 | 18.40 ± 4.24 | 16.35 ± 3.98 | 31/34 | 6.59 ± 0.93 | 6.63 ± 0.78 | 22/31 | 6.67 ± 0.94 | 6.56 ± 1.13 | 24/25 | 9.34 ± 1.03 | 8.60 ± 1.21 | 52/83 | 9.81 ± 1.09 | 9.69 ± 1.39 | 52/56 |
| HH | 14.03 ± 2.00 | 14.30 ± 2.84 | 39/35 | 17.37 ± 4.26 | 15.28 ± 4.31 | 31/35 | 5.17 ± 0.73 | 5.30 ± 0.58 | 22/31 | 5.21 ± 0.80 | 5.22 ± 0.82 | 24/25 | 5.70 ± 0.67 | 5.15 ± 0.80 | 51/85 | 6.13 ± 0.75 | 5.93 ± 0.95 | 53/56 |
| LJL | 28.31 ± 3.79 | 28.79 ± 5.71 | 39/36 | 35.33 ± 7.61 | 32.03 ± 8.70 | 29/35 | - | - | - | - | - | - | - | - | - | - | - | - |
| FTL | 11.56 ± 1.28 | 11.62 ± 1.75 | 39/36 | 13.29 ± 1.91 | 12.63 ± 2.45 | 30/35 | 3.58 ± 0.32 | 3.71 ± 0.35 | 21/30 | 3.75 ± 0.41 | 3.48 ± 0.39 | 24/25 | 4.12 ± 0.44 | 3.8 ± 0.48 | 52/85 | 4.23 ± 0.46 | 4.23 ± 0.58 | 51/55 |
| FFL | - | - | - | - | - | - | 4.8 ± 0.64 | 4.87 ± 0.46 | 22/30 | 4.88 ± 0.51 | 4.82 ± 0.47 | 24/25 | - | - | - | - | - | - |
| LFL | 17.23 ± 2.40 | 17.47 ± 3.03 | 39/36 | 20.88 ± 4.43 | 18.66 ± 4.87 | 30/35 | 4.83 ± 0.66 | 4.82 ± 0.47 | 22/31 | 4.80 ± 0.72 | 4.69 ± 0.75 | 24/25 | 6.18 ± 0.71 | 5.57 ± 0.86 | 51/83 | 6.46 ± 0.70 | 6.27 ± 0.89 | 52/55 |
| UFL | - | - | - | - | - | - | 6.13 ± 0.79 | 5.58 ± 0.66 | 22/31 | 5.84 ± 0.79 | 5.60 ± 1.01 | 24/25 | - | - | - | - | - | - |
| HTL | 20.68 ± 2.03 | 20.84 ± 2.86 | 39/35 | 23.89 ± 2.82 | 22.68 ± 3.60 | 31/35 | 6.83 ± 0.65 | 6.68 ± 0.55 | 22/29 | 6.90 ± 0.76 | 6.66 ± 0.66 | 24/25 | 4.99 ± 0.56 | 4.51 ± 0.60 | 52/84 | 5.08 ± 0.55 | 5.04 ± 0.65 | 53/56 |
| HFL | - | - | - | - | - | - | 9.1 ± 0.86 | 9.41 ± 0.91 | 21/28 | 9.42 ± 0.93 | 9.17 ± 1.06 | 24/25 | - | - | - | - | - | - |
| LHL | 21.30 ± 2.65 | 21.62 ± 3.52 | 39/35 | 25.36 ± 4.44 | 23.02 ± 5.16 | 31/34 | 6.26 ± 0.84 | 6.08 ± 0.62 | 21/30 | 6.14 ± 0.91 | 5.95 ± 0.99 | 24/25 | 7.67 ± 0.95 | 6.83 ± 1.11 | 52/84 | 8.01 ± 0.96 | 7.64 ± 1.21 | 53/56 |
| UHL | - | - | - | - | - | - | 8.03 ± 1.13 | 7.61 ± 0.89 | 22/31 | 8.01 ± 1.27 | 7.49 ± 1.26 | 24/25 | - | - | - | - | - | - |
| PH | - | - | - | - | - | - | 4.73 ± 0.9 | 4.74 ± 0.65 | 22/30 | 4.73 ± 0.75 | 4.55 ± 0.89 | 24/25 | - | - | - | - | - | - |
| PW | - | - | - | - | - | - | 5.51 ± 1.09 | 5.58 ± 0.76 | 21/31 | 5.32 ± 0.94 | 5.11 ± 1.11 | 24/25 | - | - | - | - | - | - |
| TW | - | - | - | - | - | - | 5.44 ± 1.04 | 5.62 ± 0.77 | 22/31 | 5.38 ± 0.91 | 5.33 ± 1.03 | 24/25 | - | - | - | - | - | - |
| BW | - | - | - | - | - | - | 8.81 ± 1.45 | 8.79 ± 0.94 | 20/31 | 8.15 ± 1.18 | 8.32 ± 1.18 | 24/25 | - | - | - | - | - | - |
| BL | - | - | - | - | - | - | 5.01 ± 0.72 | 5.17 ± 0.54 | 20/31 | 4.85 ± 0.65 | 4.82 ± 0.64 | 23/25 | - | - | - | - | - | - |
| Lamellae | - | - | - | - | - | - | - | - | - | - | - | - | 13.30 ± 0.66 | 12.92 ± 0.67 | 50/80 | 13.09 ± 0.57 | 13.03 ± 0.68 | 52/49 |
| Scaled mass index | 4.46 ± 0.32 | 4.44 ± 0.40 | 39/36 | 4.22 ± 0.47 | 4.44 ± 0.51 | 31/34 | 1.7 ± 0.14 | 1.74 ± 0.10 | 22/31 | 1.79 ± 0.11 | 1.71 ± 0.14 | 24/25 | 1.24 ± 0.15 | 1.03 ± 0.34 | 51/85 | 1.20 ± 0.10 | 1.21 ± 0.18 | 50/56 |
| Mite prevalence | 17.95 | 15.79 | 39/38 | 9.68 | 8.57 | 31/35 | 0 | 0 | 22/31 | 0 | 0 | 24/25 | 76.92 | 67.06 | 52/85 | 84.91 | 71.43 | 53/56 |
| Mite abundance | 1.92 ± 5.95 | 0.45 ± 1.48 | 39/38 | 1.84 ± 9.51 | 0.11 ± 0.40 | 31/35 | - | - | - | - | - | - | 12.48 ± 16.88 | 8.95 ± 16.97 | 52/85 | 24.55 ± 36.77 | 7.14 ± 8.87 | 53/56 |

## References

1. Peig, J. & Green, A. J. New perspectives for estimating body condition from mass/length data: the scaled mass index as an alternative method. *Oikos* **118**, 1883–1891 (2009).

# Supplementary Information S2

## GLMMs complete results

*Sex* came out significant for a high number of morphological traits in all three species (*P* < 0.05 for all models; Tables S2.1-S2.3), whereas *log_10_-SVL* was significantly related with all morphological traits in all of them (*P* < 0.001 in all cases; Tables S2.1-S2.3).

**Table S2.1.** GLMM results, including $\text{χ}_{\text{1}}^{\text{2}}$and *P* values, showing the effect of the invasive California kingsnake (*Lampropeltis californiae*) on each morphological and body condition traits of adult Gran Canaria giant lizards (*Gallotia stehlini*). Dependent variables are shown under *Traits* header. Models included s*nake presence*, *sex* and their interaction as fixed factors, site identity as random factor, and log_10_-SVL as a covariate. Significance is highlighted in bold letters. (-) indicate factors that were not included.

| Traits | Snakes | | Snakes X Sex | | Sex | | Log_10_-SVL | |
| --- | --- | --- | --- | --- | --- | --- | --- | --- |
|  | χ^2^ | *P* | χ^2^ | *P* | χ^2^ | *P* | χ^2^ | *P* |
| Snout-vent length | 0.11 | 0.735 | **10.25** | **0.001** | **9.58** | **0.002** | - | - |
| Head length | 0.38 | 0.538 | 1.89 | 0.169 | **91.39** | **< 0.001** | **2,974.92** | **< 0.001** |
| Head width | 2.29 | 0.130 | 0.00 | 0.967 | **11.88** | **< 0.001** | **1,852.96** | **< 0.001** |
| Head height | 0.59 | 0.442 | 1.14 | 0.287 | **7.78** | **0.005** | **1,668.29** | **< 0.001** |
| Lower jaw length | 2.13 | 0.144 | 2.23 | 0.135 | **66.08** | **< 0.001** | **2,318.76** | **< 0.001** |
| Longest fore toe length | 0.30 | 0.585 | 3.18 | 0.075 | **14.82** | **< 0.001** | **637.11** | **< 0.001** |
| Lower forelimb length | 0.21 | 0.644 | 0.82 | 0.365 | **10.82** | **0.001** | **2,934.38** | **< 0.001** |
| Longest hind toe length | 0.12 | 0.729 | 0.66 | 0.418 | **52.32** | **< 0.001** | **748.50** | **< 0.001** |
| Lower hind limb length | 0.25 | 0.616 | 0.19 | 0.663 | **55.53** | **< 0.001** | **2,325.25** | **< 0.001** |
| Scaled mass index | 0.09 | 0.759 | **4.19** | **0.041** | **6.23** | **0.013** | - | - |
| Mite prevalence | 0.08 | 0.777 | 0.02 | 0.894 | 2.04 | 0.153 | - | - |
| Mite abundance | **6.09** | **0.014** | 0.17 | 0.678 | 1.51 | 0.219 | - | - |

**Table S2.2.** GLMM results, including $\text{χ}_{\text{1}}^{\text{2}}$and *P* values, showing the effect of the invasive California kingsnake (*Lampropeltis californiae*) on each morphological and body condition traits of adult Gran Canaria skinks (*Chalcides sexlineatus*). Dependent variables are shown under *Traits* header. Models included s*nake presence*, *sex* and their interaction as fixed factors, site identity as random factor, and log_10_-SVL as a covariate. Significance is highlighted in bold letters. (-) indicate factors that were not included.

| Traits | Snakes | | Snakes X Sex | | Sex | | Log_10_-SVL | |
| --- | --- | --- | --- | --- | --- | --- | --- | --- |
|  | χ^2^ | *P* | χ^2^ | *P* | χ^2^ | *P* | χ^2^ | *P* |
| Snout-vent length | 1.33 | 0.249 | 0.24 | 0.622 | **5.09** | **0.024** | - | - |
| Head length | 2.44 | 0.119 | 0.06 | 0.807 | **23.07** | **<0.001** | **724.22** | **<0.001** |
| Head width | 1.52 | 0.217 | 0.80 | 0.371 | **37.66** | **<0.001** | **902.08** | **<0.001** |
| Head height | 0.00 | 0.954 | 0.03 | 0.864 | **10.53** | **0.001** | **465.58** | **<0.001** |
| Longest fore toe length | 3.50 | 0.062 | **7.03** | **0.008** | 0.26 | 0.609 | **73.03** | **<0.001** |
| Forefoot length | 0.26 | 0.610 | 0.04 | 0.851 | **4.26** | **0.039** | **106.71** | **<0.001** |
| Lower forelimb length | 2.17 | 0.140 | 0.13 | 0.719 | **6.49** | **0.011** | **617.68** | **<0.001** |
| Upper forelimb length | **31.10** | **<0.001** | **6.66** | **0.010** | 1.29 | 0.257 | **250.75** | **<0.001** |
| Longest hind toe length | **10.33** | **0.001** | 0.07 | 0.793 | **5.89** | **0.015** | **208.13** | **<0.001** |
| Hindfoot length | 0.49 | 0.484 | 2.87 | 0.090 | **4.01** | **0.045** | **230.75** | **<0.001** |
| Lower hind limb length | **7.75** | **0.005** | 1.50 | 0.220 | **9.40** | **0.002** | **606.94** | **<0.001** |
| Upper hind limb length | **18.52** | **<0.001** | 0.38 | 0.539 | **4.04** | **0.045** | **243.17** | **<0.001** |
| Pelvis height | 1.06 | 0.304 | 0.16 | 0.685 | **5.89** | **0.015** | **382.69** | **<0.001** |
| Pelvis width | 0.29 | 0.588 | 0.72 | 0.396 | 0.03 | 0.870 | **206.36** | **<0.001** |
| Tail width | 0.03 | 0.855 | 0.00 | 0.947 | 3.62 | 0.057 | **473.91** | **<0.001** |
| Body width | 0.09 | 0.759 | 1.96 | 0.161 | 1.47 | 0.226 | **295.66** | **<0.001** |
| Body length | 0.66 | 0.417 | 1.11 | 0.292 | 1.11 | 0.291 | **1,474.49** | **<0.001** |
| Scaled mass index | 0.73 | 0.394 | **5.16** | **0.023** | 1.03 | 0.311 | - | - |

**Table S2.3.** GLMM results—Kruskal-Wallis in the case of lamellae—including $\text{χ}_{\text{1}}^{\text{2}}$and *P* values, showing the effect of the invasive California kingsnake (*Lampropeltis californiae*) on each morphological and body condition traits of adult Boettger’s wall geckos (*Tarentola boettgeri boettgeri*). Dependent variables are shown under *Traits* header. Models included s*nake presence*, *sex* and their interaction as fixed factors, site identity as random factor, and log_10_-SVLas a covariate. Kruskal-Wallis for lamellae counts in each sex included snake presence as a grouping factor. Significance is highlighted in bold letters. (-) indicate factors that were not included in the models.

| Traits | Snakes | | Snakes X Sex | | Sex | | Log_10_-SVL | |
| --- | --- | --- | --- | --- | --- | --- | --- | --- |
|  | χ^2^ | *P* | χ^2^ | *P* | χ^2^ | *P* | χ^2^ | *P* |
| Snout-vent length | **9.57** | **0.002** | **6.49** | **0.011** | **21.01** | **<0.001** | - | - |
| Head length | 0.71 | 0.398 | 1.30 | 0.254 | **24.57** | **<0.001** | **2,656.37** | **<0.001** |
| Head width | 0.75 | 0.387 | 2.12 | 0.145 | **5.90** | **0.015** | **1,246.86** | **<0.001** |
| Head height | 0.23 | 0.635 | 1.75 | 0.186 | **5.45** | **0.020** | **837.14** | **<0.001** |
| Longest fore toe length | 0.03 | 0.870 | 0.02 | 0.883 | 2.27 | 0.132 | **358.62** | **<0.001** |
| Lower forelimb length | 0.07 | 0.798 | 2.78 | 0.096 | 0.11 | 0.742 | **1,535.80** | **<0.001** |
| Longest hind toe length | 0.59 | 0.443 | 0.90 | 0.344 | 0.37 | 0.546 | **321.80** | **<0.001** |
| Lower hind limb length | 0.66 | 0.417 | **5.96** | **0.015** | 0.56 | 0.454 | **1,542.47** | **<0.001** |
| Lamellae ♀ | **3.94** | **0.047** | - | - | - | - | - | - |
| ♂ | 0.04 | 0.834 | - | - | - | - | - | - |
| Scaled mass index | 1.39 | 0.238 | **23.85** | **<0.001** | 0.03 | 0.861 | - | - |
| Mite prevalence | 0.41 | 0.521 | 0.21 | 0.643 | 0.46 | 0.499 | - | - |
| Mite abundance | 2.78 | 0.096 | **7.25** | **0.007** | **4.81** | **0.028** | - | - |
